# Preparation and Properties of Nanocellulose from Organosolv Straw Pulp

**DOI:** 10.1186/s11671-017-2001-4

**Published:** 2017-03-31

**Authors:** V. A. Barbash, O. V. Yaschenko, O. M. Shniruk

**Affiliations:** National Technical University of Ukraine “Igor Sikorsky Kyiv Polytechnic Institute”, 37, Prospect Peremogy, Kyiv, 03056 Ukraine

**Keywords:** Wheat straw, Organosolv pulping, Nanocellulose, Hydrolysis, Ultrasound treatment

## Abstract

The object of this work is to present a study of nanocellulose preparation from organosolv straw pulp (OSP) and its properties. OSP was obtained through thermal treatment in the system of isobutyl alcohol–H_2_O–KOH–hydrazine followed by processing in the mixture of acetic acid and hydrogen peroxide for bleaching and removal of residual non-cellulosic components. We have obtained nanocellulose from OSP through acid hydrolysis with lower consumption of sulfuric acid and followed by ultrasound treatment. The structural change and crystallinity degree of OSP and nanocellulose were studied by means of SEM and XRD techniques. It has been established that nanocellulose has a density up to 1.3 g/cm^3^, transparency up to 70%, crystallinity degree 72.5%. The TEM and AFM methods shown that nanocellulose have diameter of particles in the range from 10 to 40 nm. Thermogravimetric analysis confirmed that nanocellulose films have more dense structure and smaller mass loss in the temperature range 220–260 °C compared with OSP. The obtained nanocellulose films had high Young’s modulus up to 11.45 GPa and tensile strength up to 42.3 MPa. The properties of obtained nanocellulose from OSP exhibit great potential in its application for the preparation of new nanocomposite materials.

## Background

Nanocellulose is steadily gaining attention since this material is a renewable alternative to artificial polymers [1]. Research and development of materials obtained from renewable natural sources has been the focus of attention in various engineering applications [2]. The use of different kinds of lignocellulosic materials has great potential for production of biocomposites, which are applied in optoelectronic devices, packaging, and building [3].

Nanocellulose belongs to a group of nanomaterials consisting of the nanosized cellulose particles. Characteristics of nanocellulose particles depend on the properties of plant raw materials and methods used for their production [4]. Nanocellulose prepared from renewable lignocellulose materials has improved mechanical properties, such as high strength, flexibility, high surface area-to-volume ratio, and high aspect ratio (fiber length to width ratio) [5, 6]. The cellulose nanomaterials exhibit excellent properties, such as high elastic modulus, high specific surface area, optical transparency, low thermal expansion coefficient, and chemical reactivity [2]. Nanocellulose often replaces such well-known material, as glass and certain polymers, which are not biodegradable at ambient conditions, in order to create new specific nanocomposites, adsorbents, and functional materials for the electrodes in chemical sources of power and optoelectronic devices [7–9]. It is also used for production of biodegradable plastics and paper with special characteristics [10]. Nanocellulose finds its application in nanocomposites [11–14], to increase their strength and thermal resistance [15] and to stabilize the emulsions [16], in preparation of bio-basic films [17].

In world practice, there are methods of obtaining nanocellulose from kanaf [18], oat husk [19], coconut fibers [20], and other cellulose-containing materials [21–23]. During the processing of grain and industrial crops, the stalks and fibers of plants are formed which can be used as an alternative to wood in production of cellulose. Wheat straw, millions of which are annually produced in agriculturally developed countries, can also be attributed to promising representatives of non-wood plant raw materials for obtaining cellulose.

In the global practice of pulp and paper industry, the dominating technologies to obtain cellulose are sulfate and sulfite methods, which lead to environmental pollution. Increased environmental requirements to the quality of wastewater and gas emissions of industrial enterprises requires the development of new technologies for processing of plant raw materials with the use of organic solvents [24, 25]. For example, peracetic acid is a strong oxidizing agent with excellent bleaching properties. It is an environmentally safe alternative for bleaching because it is a total chlorine-free process resulting in less damage to the fiber [26].

We have already demonstrated the possibility of obtaining straw pulp by means of organosolv delignification in the system of isobutyl alcohol–H_2_O–KOH–hydrazine, which makes it possible to reuse the organic component and waste cooking liquor without regeneration [27]. At the same time the waste liquor is divided into two layers: the upper organic solvent layer and the lower aqueous layer to which has moved the bulk of soluble minerals and organic substances from plant raw material (lignin, hemicelluloses, and extractives). The use of potassium and nitrogen compounds in the cooking liquor allows the use of waste liquor in the manufacture of fertilizers. Previously, we have also obtained nanofibrillated cellulose (CNF) from the air-dry-bleached softwood sulfate pulp with the use of mechanochemical treatment [28]. Mechanochemical treatment was performed with the use of a milling equipment common for pulp and paper industry. In this research, to reduce energy consumption for preparation of nanocellulose, we used organosolv straw pulp (OSP) which was never dried after cooking and bleaching. Never-dried cellulose is better than once-dried samples because the latter are known to irreversibly lose surface accessibility as a result of drying. Using of never-dried cellulose does not require consumption of energy for drying and grinding since dried cellulose fibers lose the ability to swell and percolate due to irreversible cornification. The application of wet cellulose enables better percolation of acid into cellulose fibers.

We investigated the possibility of obtaining nanocellulose from never-dried OSP using only sulfuric acid hydrolysis and ultrasound treatment, and we defined the mechanical and thermal properties of nanocellulose.

## Methods

In order to obtain pulp, stalks of wheat straw from Kiev region harvested in 2015 were used. Averaged chemical composition relative to absolutely dry raw material (a.d.r.m.) was 44.2% of cellulose, 18.6% of lignin, 25.2% of pentosans, 4.2% of ash, 4.9% of resin, fats and waxes, and 71.8% of holocellulose. Chemical composition of wheat straw stalks was identified according to standard methods [29]. For each, parameters were made two parallel measurements and the resulting mean value was given in the text. Before research, the raw material was ground to 2–5 mm and stored in desiccator for maintenance of constant humidity and chemical composition.

Cooking of straw stalks in the system isobutyl alcohol–H_2_O–KOH–hydrazine was carried out according to the procedure described in [27]. The received organosolv pulp had the following quality indicators: yield of pulp—49%, residual lignin—1.1%, ash—1.63%, pentosans—0.93% to a.d.r.m, whiteness—51%.

In order to remove residual lignin and carried out partial hydrolysis of hemicellulose, we additionally carried out thermochemical treatment of OSP using acetic acid and hydrogen peroxide in a volume ratio of 70:30% with the catalyst–sulfuric acid which was 15% to a.d.r.m mass. Treatment with the mixture was carried out 180 min at a temperature of 95 ± 2 °C. We received the bleached OSP with ash content of 0.2%, lignin—less than 0.2%, degree of polymerization—460, whiteness—83%, and used it for preparation of nanocellulose.

Hydrolysis of never-dried bleached OSP was carried out by means of sulfuric acid with concentration of 43%, at the liquid to solid ratio 10:1, at temperature 20 and 60 °C during 30 and 60 min. The hydrolyzed cellulose was rinsed with distilled water three times by means of centrifugation at 8000 rev/min and subsequent dialysis until reaching neutral pH. Ultrasound treatment of hydrolyzed cellulose was performed using ultrasound disintegrator UZDN-A (SELMI, Ukraine) with 22 kGz for 30 min. The cellulose dispersion was placed in an ice bath to prevent overheating during treatment. Eventually, the suspension had the form of a homogenous gel-like dispersion.

The prepared suspensions were poured into Petri dishes and dried in air at a room temperature to obtain nanocellulose films. Their density was determined according to the ISO 534:1988. The degree of polymerization was determined according to ISO 5351 by the viscosity of the samples dissolved in copper ethylene-diamine solution. Scanning electron microscope (SEM) analysis was performed with PEM–106I (SELMI, Ukraine) microscope to observe the morphology of OSP and CNF films. Transparency of the nanocellulose films was determined by electron absorption spectra, which were registered in regions from 200 to 1100 nm. Electron absorption spectra of the nanocellulose films in UV and in visible and near infrared regions were registered on two-beam spectrophotometer 4802 (UNICO, USA) with resolution of 1 nm.

Transmission electron microscopy (TEM) images were obtained using electron microscope TEM125K (SELMI, Ukraine) operating at a potential of 100 kV. A dilute CNF suspension (0.1 wt%) was dropped onto a thin scaffoldings Lacey Formvar/Carbon, 400 mesh, copper approx. grid hole size 42 μm (TED PELLA, Inc, USA). Topographical characterization of nanocellulose samples was investigated using atomic force microscopy (AFM), and the measurements were accomplished with Si cantilever, operating in the tapping mode on the device Solver Pro M, NT-MDT, Russia. The scanning speed and area were 0.6 line/s and 2 × 2 μm^2^, respectively. Before AFM investigation, dilute nanocellulose suspensions with the concentration of 0.01 wt% were ultrasonically treated for 10 min. Subsequently, one drop of CNF dispersion for sample was injected onto a freshly cleaned glass-ceramic and air dried at room temperature.

X-ray diffraction patterns of different cellulose samples were obtained by Ultima IV diffractometer (Rigaku, Japan). The method proposed in [30] was used to determine the crystallinity degree (CD) of the samples, in terms of which CD = (*I*_200_ − *I*_am_)/*I*_200_ × 100%, where *I*_200_ is an intensity of (200) reflex about 22.5°, and *I*_am_ is an intensity of amorphous scattering at 18.5°.

The thermal degradation behavior of cellulose and CNF samples was explored by heating using Netzsch STA-409 thermoanalyzer. The samples were heated at a rate of 5 °C/min, from 25 to 450 °C.

Tensile properties of the nanocellulose films were measured at controlled temperature (23 ± 1 °C) and humidity (50 ± 2%) according to ISO 527-1. Tension tests were performed at a crosshead speed of 0.5 mm/min on the TIRAtest-2151 (Germany) instrument equipment with a 2-N load stress. For testing, test strips with 10 ± 2 mm width and 25 ± 5 mm long were used. The data reported are tensile strength and Young’s modulus. Each composition was tested with a minimum of five specimens to extract an average and standard deviation for each property.

## Results and Discussion

**Fig. 1:**
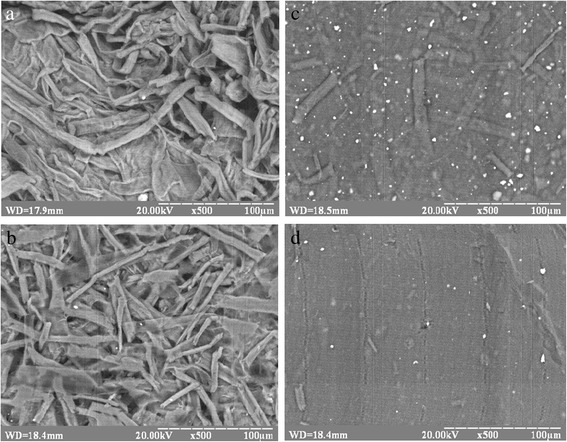
SEM images of organosolv straw pulp: the initial (**a**), after hydrolysis (**b**), after sonication (**c**), and after hydrolysis and sonication

Morphology of initial OSP and obtained nanocellulose films were studied by means of SEM. Figure 1 presents electron micrographs of the surfaces of initial organosolv straw pulp and nanocellulose films prepared after hydrolysis and sonication of OSP water suspensions. As shown in Fig. 1, the length of the fibers of initial OSP is more than 100 μm and width is from 10 to 20 μm (Fig. 1a). Hydrolysis of bleached OSP leads to a significant decrease in the length and diameter of its fibers (Fig. 1b). Sonication of hydrolyzed cellulose significantly reduces the fiber length to 50 μm. As shown by SEM micrographs, the ultrasound treatment of OSP forms a film with a dense structure (Fig. 1c). These films were not optically transparent, but translucent. Further sonication of cellulose after hydrolysis facilitates formation of dense films separate fibers of which are not visualized (Fig. 1d). Besides, the obtained films are optically transparent. The claim about dense homogeneous structure is due to the absence of individual fibers in SEM images after cellulose treatment by hydrolysis and ultrasound and also by the obtained transparent nanocellulose films. This result means that the cavities in the sheet were almost completely removed. The resulting dried films were optically transparent and looked like plastic films, indicating that the light scattering in the bulk sheet was completely suppressed. The structure of obtained films was similar to the structure of the film received from nanofibrilic cellulose in article Nogi et al [31]. The white flecks in Fig. 1c, d are charges on the surface of the film because cellulose is not a conductive material.

**Fig. 2:**
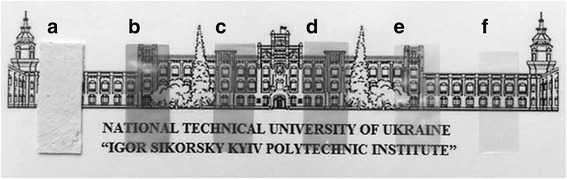
**a** Comparison of transparent strips made from initial OSP. **b** OSP after ultrasound treatment. CNF films prepared after hydrolysis by sulfuric acid of concentration 43% at (**c**) 20 °C, 30 min; **d** 20 °C, 60 min; **e** 60 °C, 30 min; **f** and 60 °C, 60 min. The duration of ultrasound treatment all patterns was 30 min

**Fig. 3:**
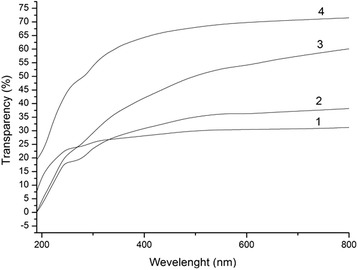
Electron absorption spectra of the nanocellulose films prepared from OSP dispersion after hydrolysis by sulfuric acid of concentration 43% and ultrasound treatment 30 min for different temperature and duration: 20 °C, 30 min (*1*); 20 °C, 60 min (*2*); 60 °C, 30 min (*3*) and 60 °C, 60 min (*4*)

Changes in the look of organosolv pulp and CNF films according to the stages of treatment are presented in Fig. 2. Comparison of the strips shows that films made from OSP after sonication and CNF films prepared after hydrolysis and ultrasound treatment indeed is transparent. These results indicate that the chemical treatment and sonication of organosolv straw pulp leads to the formation of homogeneous nanocellulose films with high transparency up to 69.8% at the wavelength of 600 nm (Fig. 3).

**Fig. 4:**
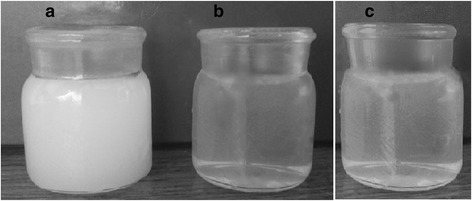
Photographs of the nanocellulose vials prepared after hydrolysis of OSP by sulfuric acid of concentration 43% at 60 °C and 60 min: without ultrasound treatment (**a**) and after ultrasound treatment (**b**), and after 3 months of storage (**c**)

Nanocellulose prepared after hydrolysis and ultrasound treatment of OSP had homogeneous and stable nanocellulose suspension. The nature of stabilization of the colloidal suspension is explained by the presence of charged groups on the surface of nanocellulose, which are formed by the interaction of cellulose with sulfate acid due to the esterification reaction. As proof of the stability nanocellulose suspensions, we give its images immediately after the preparation and after a prolonged storage time (Fig. 4). There was no sedimentation of nanocellulose particles when stored at room temperature for extended period. Figure 4 presents photographs of vials of nanocellulose prepared after hydrolysis of OSP by sulfuric acid of concentration 43% at 60 °C and 60 min without ultrasound treatment, immediately after ultrasound treatment and after 3 months of storage (Fig. 4c). Such stabilization of nanocellulose suspensions is evidenced by article [32].

The decrease of the cellulose particles size and the increase of its dispersity were assessed by measuring the changes in the degree of polymerization (DP). Thus, DP of the initial organosolv straw pulp was 460; DP of OSP after sonication was 390; DP of nanocellulose after hydrolysis with 43% sulfuric acid was 210; DP of nanocellulose after hydrolysis and sonication was 105. From the date, it can be see that hydrolysis of OSP reduces the degree of polymerization more intensively than ultrasound. The joint action of sulfuric acid and sonication leads to a substantial reduction of the cellulose macromolecules.

**Fig. 5:**
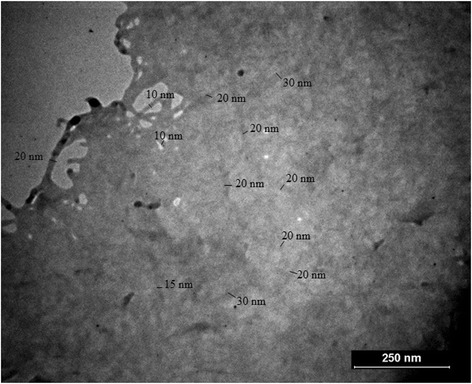
TEM images of nanocellulose prepared by hydrolysis with H_2_SO_4_ concentration 43% at 40 °C and ultrasound treatment for 30 min

**Fig. 6:**
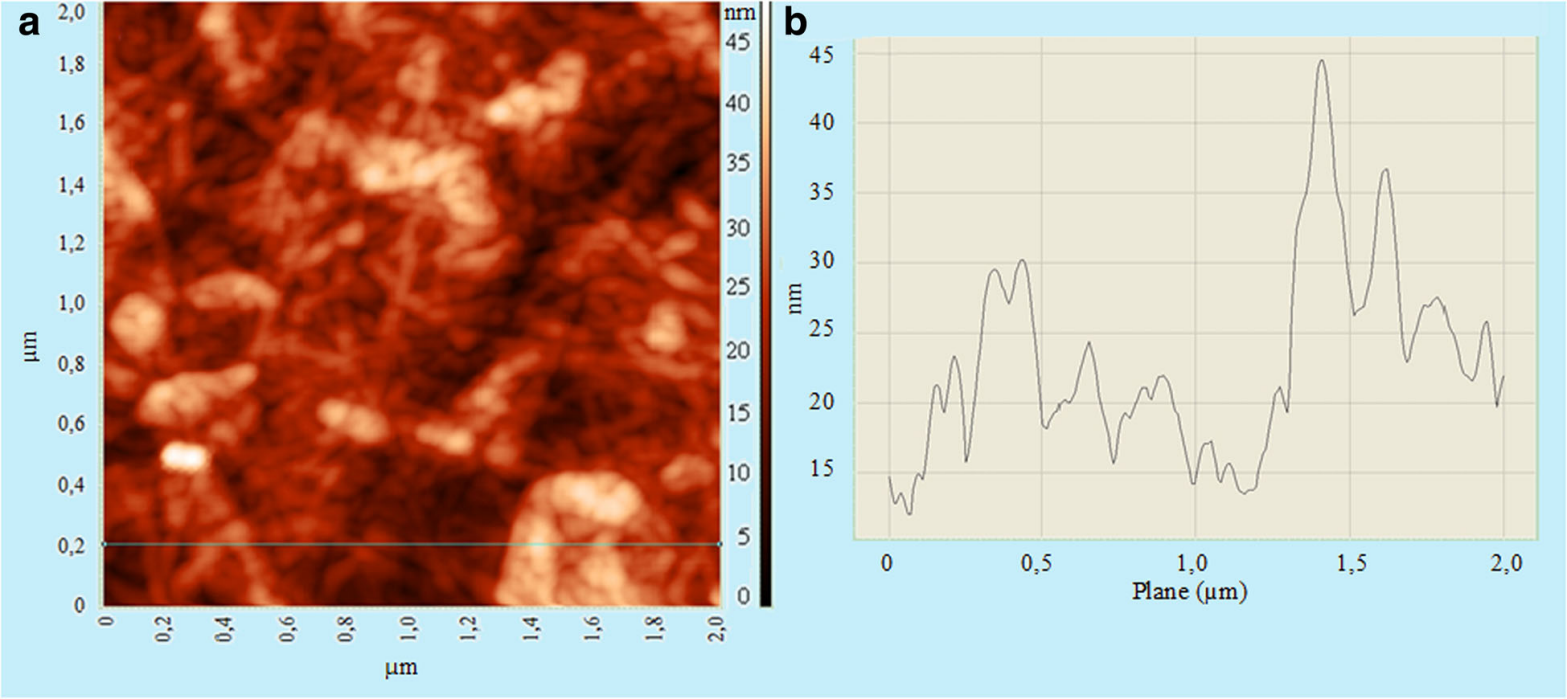
The AFM images of organosolv straw nanocellulose film height (**a**) and amplitude (**b**) tapping mode

In order to assess the changes in the particles size after hydrolysis and sonication, we examined the morphology of nanocellulose samples with the use of TEM and AFM (Figs. 5 and 6). As can be seen from Fig. 5, nanocellulose was obtained after hydrolysis with sulfuric acid and sonication is nanofibrillated cellulose (CNF) with multilayer structure. Nanofibers form a delicate mesh as a result of interaction between the particles of nanocellulose. In addition, it was experimentally found that the diameter of separate nanocellulose particles ranges from 10 to 40 nm, and their length is up to several micrometers. These nanofibers possess a high aspect ratio. These dimensions correspond to data obtained by AFM (Fig. 6b). As shown in Fig. 6a, nanocellulose particles aggregated and interlaced. The diameter of separate nanofibers is within the range to 40 nm (Fig. 6). This result was confirmed by CNF dimension data obtained due to the mechanochemical method for bleached softwood sulfate pulp [28].

**Fig. 7:**
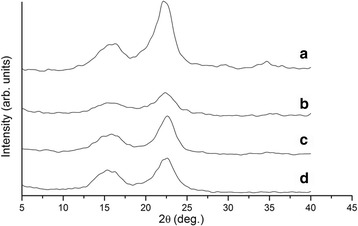
X-ray diffraction patterns of the organosolv straw pulps: the initial (**a**), after sonication (**b**), after hydrolysis (**c**), and after hydrolysis and sonication (**d**)

We have also investigated the change in the ratio of amorphous and crystalline parts of OSP during its chemical and physical treatment. The analysis of X-ray diffraction patterns of initial OSP (Fig. 7a), after sonication (Fig. 7b), after hydrolysis (Fig. 7c), and after hydrolysis and sonication (Fig. 7d) was carried out, and its degree of crystallinity was calculated. In the structure of initial organosolv straw pulp, the peaks were observed at 16°, 23°, and 34°. These peaks are common for type I cellulose [26]. The crystallinity degree of initial OSP is 72.5%, which is higher than that for organosolv pulp produced by Sánchez et al. [3], and is significantly reduced to 57.8% during sonication due to partial destruction of crystalline areas of macromolecules under the high energy of ultrasound. At the same time, hydrolysis of initial OSP increases the crystallinity to 76.3% due to removal of amorphous parts of cellulose. The additional sonication after hydrolysis leads to partial degradation of crystalline parts of macromolecules and to a slight decrease in the crystallinity degree of nanocellulose to 72.5%.

**Fig. 8:**
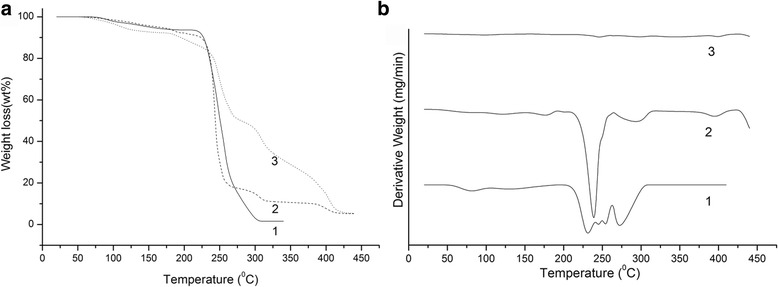
Gravimetric (**a**) and differential (**b**) curves of thermal analysis: pulp after the first stage of pulping in isobutanol (*1*); pulp after the second stage of thermochemical treatment in the mixture of peracetic acid (*2*); nanocellulose film (*3*)

The increase of crystallinity parts after treatment of the initial OSP is confirmed by the thermogravimetric analysis (TGA) of OSP and nanocellulose films (Fig. 8). Figure 8 shows the change of the thermal stability of OSP samples after the first stage of pulping in isobutanol (curve 1), after the second stage of thermochemical treatment in the mixture of peracetic acid (curve 2), and of nanocellulose films after hydrolysis with 43% sulfuric acid and sonication (curve 3). As seen from the thermogravimetric curves (Fig. 8), weight loss of all OSP samples starts at a temperature about 80 °C, which is due to evaporation of residual moisture from the fiber. The main weight loss after the first stage of pulping under thermal degradation starts at temperatures between 220–240 °C and continues up to 300 °C. Curve 2 is characterized by weight loss of about 60% in the temperature range 220–260 °C and continues up to 400 °C as the second stage of pulping with the elements of hydrolysis increases the amount of crystalline part of cellulose, and it strengthens the hydrogen bonds between cellulose macromolecules. Destruction of nanocellulose films (curve 3) has a different nature. Nanocellulose films lose up to 10% of weight at a temperature of 160 °C, and at 240 °C, another 10% of weight is lost. For nanocellulose films the abrupt weight loss is not typical in the temperature range 220–260 ° C, it happens gradually as the temperature increases up to 400 °C. It can be explained by the fact that during the chemical treatment and ultrasonic homogenization a dense structure between pulp molecules is formed.

**Fig. 9:**
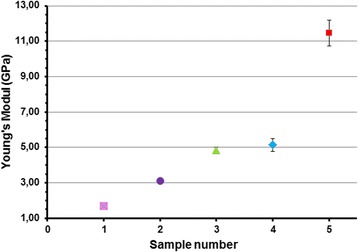
The Young’s modulus of the organosolv straw pulp and nanocellulose films (sample number correspond to numeration in Table 1).

In this research, mechanical parameters of initial OSP and nanocellulose films prepared after hydrolysis of OSP at different temperature and duration and additional sonication during 30 min were measured (Table 1 and Fig. 9). The average values and standard deviations for independent measurement of different samples were shown in Table 1. Figure 9 shows how the Young’s modulus of OSP and nanocellulose films depend on the conditions of treatment. As follows from Table 1 and Fig. 9, with the increase of temperature and duration of hydrolysis, such indicators as density, tensile strength, Young’s modulus, and transparency are also increasing. With the intensification of cellulose hydrolysis condition, the resistance of nanocellulose films to deformation increases compared to that of non-hydrolyzed cellulose. It happens due to the formation of a dense structure of CNF film through hydrolysis of amorphous cellulose part and the increase in ordered crystalline parts of nanocellulose, which promotes the formation of stronger bonds between cellulose macromolecules. Therefore, Young’s modulus increases from 1.7 to 11.45 GPa, which corresponds to the data obtained by Josset et al. [33]. Authors show that Young’s modulus values for films generated from nanofibrillated wheat straw cellulose are up to 6 GPa. Thus, the obtained cellulose has the necessary properties and can be used as a potential raw material for the production of new nanocomposite materials.

**Table 1:** The dependence of the properties of organosolv straw pulp and nanocellulose films on hydrolysis conditions and duration of the ultrasound treatment for 30 min

No. of the sample	Temperature, °C	Duration of hydrolysis, min	Density, g/cm^3^	Tensile strength, MPa	Elongation at break, %	Transparency, %
1	–	–	0.8 ± 0.038	30.2 ± 1.34	1.8 ± 0.06	–
2	20	30	0.91 ± 0.04	37.5 ± 0.6	1.2 ± 0.06	30.5
3	20	60	0.98 ± 0.04	50.0 ± 0.9	1.04 ± 0.05	36.0
4	60	30	1.1 ± 0.05	41.3 ± 3.87	0.75 ± 0.05	54.0
5	60	60	1.3 ± 0.03	42.3 ± 1.87	0.37 ± 0.02	69.8

## Conclusions

In this research work, the organosolv pulp and nanocellulose were prepared from the wheat straw, one of the most abundant sources of cellulose for countries with limited supply of wood. Organosolv pulp was obtained from stalks of wheat straw by delignification in isobutanol–H_2_O–KOH–hydrazine solution, which is an effective system for removal of lignin and hemicellulose components. Thermochemical treatment of organosolv pulp using acetic acid and hydrogen peroxide was carried out for remove residual lignin and partial hydrolysis of hemicellulose. Organosolv pulp is an interesting low-cost starting material for nanocellulose production with using of sulfuric acid and sonication. The advantages of this method of nanocellulose preparation consist in using low concentration of sulfuric acid (43%) before ultrasonic treatment of organosolv wheat straw pulp. To form the idea about the structure and properties of OSP nanocellulose, a wide variety of measurement methods (SEM, TEM, AFM, XRD, TGA and DTA, mechanical research) were used. It was found out that by using the method of acid hydrolysis and ultrasound treatment of OSP it is possible to obtain nanocellulose with density up to 1.3 g/cm^3^, transparency up to 70%, crystallinity degree 72.5%. The TEM and AFM methods show that nanocellulose have diameter of particles in the range from 10 to 40 nm. Thermogravimetric analysis confirmed that nanocellulose films have more dense structure and smaller mass loss in the temperature range 220–260 °C compared with OSP. The obtained nanocellulose films had high Young’s modulus up to 11.45 GPa and tensile strength up to 42.3 MPa. The properties of obtained nanocellulose from OSP exhibit great potential in its application for the preparation of new nanocomposite materials, for example for production of biocomposites, which are applied in optoelectronic devices, packaging, and building.
